# {6,6′-Dieth­oxy-2,2′-[2,2-dimethyl­propane-1,3-diylbis(nitrilo­methyl­idyne)]diphenolato}(2-eth­oxy-6-formyl­phenolato)cobalt(III)–ethanol–water (1/1/1)

**DOI:** 10.1107/S1600536810007622

**Published:** 2010-03-06

**Authors:** Reza Kia, Hadi Kargar, Karim Zare, Islam Ullah Khan

**Affiliations:** a Department of Chemistry, Science and Research Branch, Islamic Azad University, Tehran, Iran; b Department of Chemistry, School of Science, Payame Noor University (PNU), Ardakan, Yazd, Iran; c Materials Chemistry Laboratory, Department of Chemistry, GC University, Lahore 54000, Pakistan

## Abstract

The asymmetric unit of the title compound, [Co(C_23_H_28_N_2_O_4_)(C_9_H_9_O_3_)]·C_2_H_5_OH·H_2_O, comprises one complex mol­ecule, a water mol­ecule of crystallization and an ethanol mol­ecule of crystallization, which is disordered over two positions with a ratio of refined site occupancies of 0.567 (10):0.433 (10). The Co^III^ ion is in a slightly distorted octa­hedral geometry involving an N_2_O_2_ atom set of the tetra­denate Schiff base ligand and two O atoms of 2-eth­oxy-6-formyl­phenolate. The H atoms of the water mol­ecule act as donors in the formation of bifurcated inter­molecular O—H⋯(O,O) hydrogen bonds with the O atoms of the hydr­oxy and eth­oxy groups with *R*_1_^2^(5) ring motifs, which may influence the mol­ecular conformation. The crystal structure is further stabilized by inter­molecular O—H⋯O and C—H⋯O inter­actions.

## Related literature

For hydrogen-bond motifs, see: Bernstein *et al.* (1995 ▶). For bond-length data, see: Allen *et al.* (1987 ▶). For background to Schiff base–metal complexes, see: Granovski *et al.* (1993 ▶); Blower *et al.* (1998 ▶); Elmali *et al.* (2000 ▶).
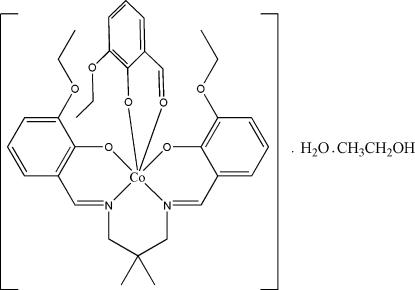


## Experimental

### 

#### Crystal data

[Co(C_23_H_28_N_2_O_4_)(C_9_H_9_O_3_)]·C_2_H_6_O·H_2_O
*M* *_r_* = 684.65
Monoclinic, 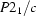

*a* = 13.2827 (17) Å
*b* = 14.0158 (17) Å
*c* = 19.602 (2) Å
β = 106.491 (7)°
*V* = 3499.1 (8) Å^3^
*Z* = 4
Mo *K*α radiation
μ = 0.54 mm^−1^
*T* = 298 K
0.42 × 0.21 × 0.15 mm

#### Data collection

Bruker SMART APEXII CCD area-detector diffractometer
Absorption correction: multi-scan (*SADABS*; Bruker, 2005 ▶) *T*_min_ = 0.804, *T*_max_ = 0.923
69893 measured reflections
6159 independent reflections
3652 reflections with *I* > 2σ(*I*)
*R*_int_ = 0.115

#### Refinement

*R*[*F* ^2^ > 2σ(*F* ^2^)] = 0.051
*wR*(*F* ^2^) = 0.138
*S* = 1.05
6159 reflections
424 parameters
6 restraints
H-atom parameters constrained
Δρ_max_ = 0.35 e Å^−3^
Δρ_min_ = −0.38 e Å^−3^

### 

Data collection: *APEX2* (Bruker, 2005 ▶); cell refinement: *SAINT* (Bruker, 2005 ▶); data reduction: *SAINT*; program(s) used to solve structure: *SHELXTL* (Sheldrick, 2008 ▶); program(s) used to refine structure: *SHELXTL*; molecular graphics: *SHELXTL*; software used to prepare material for publication: *SHELXTL* and *PLATON* (Spek, 2009 ▶).

## Supplementary Material

Crystal structure: contains datablocks global, I. DOI: 10.1107/S1600536810007622/jh2132sup1.cif

Structure factors: contains datablocks I. DOI: 10.1107/S1600536810007622/jh2132Isup2.hkl

Additional supplementary materials:  crystallographic information; 3D view; checkCIF report

## Figures and Tables

**Table 1:** Hydrogen-bond geometry (Å, °)

*D*—H⋯*A*	*D*—H	H⋯*A*	*D*⋯*A*	*D*—H⋯*A*
O1*W*—H1*WA*⋯O1	0.85	2.51	3.182 (5)	137
O1*W*—H1*WA*⋯O4	0.85	2.15	2.936 (5)	154
O1*W*—H1*WB*⋯O2	0.85	2.21	2.883 (5)	136
O1*W*—H1*WB*⋯O5	0.85	2.18	2.952 (5)	151
O7*A*—H7*A*⋯O1*W*	0.82	2.10	2.899 (19)	164
C8—H8*C*⋯O3	0.97	2.31	2.829 (5)	113

**Fig. 1.**
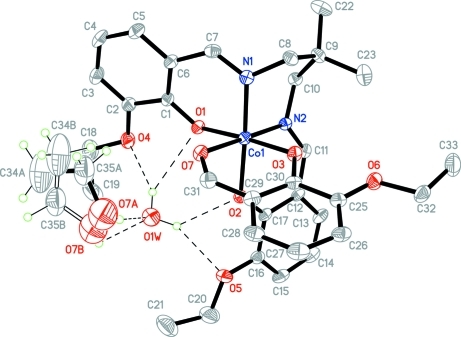
The asymmetric unit of the title compound, showing 30% probability displacement ellipsoids and the atomic numbering. All H atoms except those of water and ethanol molecules were omitted for clarity. Intramolecular hydrogen bonds are drawn as dashed lines.

## supplementary crystallographic information

### Comment

Schiff base complexes are one of the most important stereochemical models in transition metal coordination chemistry, with the ease of preparation and structural variations (Granovski *et al.,* 1993). Metal derivatives of the Schiff bases have been studied extensively, and they play a major role in both synthetic and structurel research (Elmali *et al.,* 2000; Blower *et al.,* 1998). The structure of the title compound was determined to clarify the identity of the synthesis product.

The asymmetric unit of the title compound, Fig. 1, [Co(C_32_H_37_N_2_O_7_)]. C_2_H_6_O. H_2_O, comprises a unit of the complex, a water molecule of crystallization and an ethanol of crystallization. The H atoms of the water molecule act as donors in the formation of bifurcated O—H···(O,O) intermolecular hydrogen bonds with the O atoms of the hydroxy and ethoxy groups with R^2^_1_(5) ring motifs (Bernstein *et al.,* 1995) which may influence the molecular conformation. The crystal structure is further stabilized by the intermolecular C—H···O and O—H···O interactions (Table 1).

### Experimental

The title compound was synthesized by adding 6,6'-Diethoxy-2,2'- [2,3-dimethyl-propylenebis(nitrilomethylidyne)]-diphenol (2 mmol) to a solution of CoCl_2_. 6 H_2_O (2 mmol) in ethanol (30 ml). The mixture was refluxed with stirring for half an hour. The resultant red solution was filtered. Brown single crystals of the title compound suitable for *X*-ray structure determination were recrystallized from ethanol by slow evaporation of the solvents at room temperature over several days.

### Refinement

The H atoms of the water molecule were located in a difference Fourier map and constrained to refine with the parent atom with U_iso_(H) = 1.5 U_eq_(O). The H atoms of the ethanol molecules were positioned geometrically and constrained to refine with the parent atoms with U_iso_(H) = 1.5 U_eq_(O). The rest of the H atoms were positioned geometrically and refined using a riding model with U_iso_(H) = 1.2 or 1.5 U_eq_(C). Distant restraints were applied to the ethanol molecules.

### Crystal data

**Table:**

[Co(C_23_H_28_N_2_O_4_)(C_9_H_9_O_3_)]·C_2_H_6_O·H_2_O	*F*(000) = 1448
*M**_r_* = 684.65	*D*_x_ = 1.300 Mg m^−^^3^
Monoclinic, *P*2_1_/*c*	Mo *K*α radiation, λ = 0.71073 Å
Hall symbol: -P 2ybc	Cell parameters from 6054 reflections
*a* = 13.2827 (17) Å	θ = 2.6–18.8°
*b* = 14.0158 (17) Å	µ = 0.54 mm^−^^1^
*c* = 19.602 (2) Å	*T* = 298 K
β = 106.491 (7)°	Block, brown
*V* = 3499.1 (8) Å^3^	0.42 × 0.21 × 0.15 mm
*Z* = 4	

### Data collection

**Table:**

Bruker SMART APEXII CCD area-detector diffractometer	6159 independent reflections
Radiation source: fine-focus sealed tube	3652 reflections with *I* > 2σ(*I*)
graphite	*R*_int_ = 0.115
φ and ω scans	θ_max_ = 25.0°, θ_min_ = 2.2°
Absorption correction: multi-scan (*SADABS*; Bruker, 2005)	*h* = −15→15
*T*_min_ = 0.804, *T*_max_ = 0.923	*k* = −16→16
69893 measured reflections	*l* = −23→23

### Refinement

**Table:**

Refinement on *F*^2^	Primary atom site location: structure-invariant direct methods
Least-squares matrix: full	Secondary atom site location: difference Fourier map
*R*[*F*^2^ > 2σ(*F*^2^)] = 0.051	Hydrogen site location: inferred from neighbouring sites
*wR*(*F*^2^) = 0.138	H-atom parameters constrained
*S* = 1.05	*w* = 1/[σ^2^(*F*_o_^2^) + (0.0534*P*)^2^ + 2.3725*P*] where *P* = (*F*_o_^2^ + 2*F*_c_^2^)/3
6159 reflections	(Δ/σ)_max_ = 0.001
424 parameters	Δρ_max_ = 0.35 e Å^−^^3^
6 restraints	Δρ_min_ = −0.38 e Å^−^^3^

### Special details

**Table:**

Geometry. All esds (except the esd in the dihedral angle between two l.s. planes) are estimated using the full covariance matrix. The cell esds are taken into account individually in the estimation of esds in distances, angles and torsion angles; correlations between esds in cell parameters are only used when they are defined by crystal symmetry. An approximate (isotropic) treatment of cell esds is used for estimating esds involving l.s. planes.
Refinement. Refinement of F^2^ against ALL reflections. The weighted R-factor wR and goodness of fit S are based on F^2^, conventional R-factors R are based on F, with F set to zero for negative F^2^. The threshold expression of F^2^ > 2sigma(F^2^) is used only for calculating R-factors(gt) etc. and is not relevant to the choice of reflections for refinement. R-factors based on F^2^ are statistically about twice as large as those based on F, and R- factors based on ALL data will be even larger.

### Fractional atomic coordinates and isotropic or equivalent isotropic displacement parameters (Å^2^)

**Table:**

	*x*	*y*	*z*	*U*_iso_*/*U*_eq_	Occ. (<1)
Co1	0.08603 (4)	0.66689 (3)	0.10882 (3)	0.03716 (18)	
O1	0.16611 (19)	0.55376 (17)	0.11696 (13)	0.0400 (6)	
O2	0.1630 (2)	0.71434 (17)	0.04880 (13)	0.0420 (7)	
O3	0.0100 (2)	0.78205 (17)	0.09545 (13)	0.0440 (7)	
O4	0.3340 (2)	0.4494 (2)	0.13168 (16)	0.0633 (8)	
O5	0.2931 (2)	0.7862 (2)	−0.01411 (17)	0.0634 (9)	
O6	−0.1080 (2)	0.92553 (19)	0.04635 (16)	0.0558 (8)	
N1	0.0146 (3)	0.6214 (2)	0.17440 (16)	0.0411 (8)	
N2	−0.0255 (2)	0.6182 (2)	0.03404 (16)	0.0360 (7)	
O7	0.1986 (2)	0.71601 (19)	0.18559 (13)	0.0471 (7)	
C1	0.1982 (3)	0.5046 (3)	0.1762 (2)	0.0401 (9)	
C17	0.1290 (3)	0.7202 (2)	−0.0207 (2)	0.0390 (10)	
C30	0.0521 (3)	0.8646 (3)	0.1149 (2)	0.0409 (10)	
C10	−0.1038 (3)	0.5570 (3)	0.0523 (2)	0.0441 (10)	
H10A	−0.0682	0.5028	0.0794	0.053*	
H10B	−0.1517	0.5328	0.0087	0.053*	
C12	0.0297 (3)	0.6901 (2)	−0.0623 (2)	0.0400 (10)	
C6	0.1477 (3)	0.5041 (3)	0.2303 (2)	0.0463 (10)	
C11	−0.0402 (3)	0.6393 (2)	−0.0321 (2)	0.0399 (10)	
H11	−0.1032	0.6192	−0.0634	0.048*	
C32	−0.1803 (4)	1.0022 (3)	0.0196 (3)	0.0634 (13)	
H32A	−0.1557	1.0412	−0.0132	0.076*	
H32B	−0.1870	1.0422	0.0585	0.076*	
C16	0.1978 (4)	0.7582 (3)	−0.0578 (2)	0.0477 (11)	
C25	−0.0104 (4)	0.9476 (3)	0.0882 (2)	0.0450 (10)	
C8	−0.0929 (3)	0.6532 (3)	0.1644 (2)	0.0487 (11)	
H8C	−0.0960	0.7222	0.1607	0.058*	
H8B	−0.1159	0.6348	0.2053	0.058*	
C7	0.0531 (3)	0.5571 (3)	0.2221 (2)	0.0492 (11)	
H7	0.0154	0.5441	0.2544	0.059*	
C29	0.1529 (3)	0.8801 (3)	0.1619 (2)	0.0471 (10)	
C2	0.2861 (3)	0.4432 (3)	0.1853 (2)	0.0527 (11)	
C13	0.0010 (4)	0.6986 (3)	−0.1375 (2)	0.0547 (12)	
H13	−0.0654	0.6791	−0.1642	0.066*	
C28	0.1908 (4)	0.9753 (3)	0.1800 (2)	0.0575 (12)	
H28	0.2571	0.9853	0.2114	0.069*	
C14	0.0689 (4)	0.7348 (3)	−0.1707 (2)	0.0604 (13)	
H14	0.0494	0.7397	−0.2201	0.073*	
C9	−0.1676 (3)	0.6083 (3)	0.0956 (2)	0.0484 (11)	
C31	0.2158 (3)	0.8032 (3)	0.1952 (2)	0.0521 (11)	
H31	0.2784	0.8194	0.2288	0.063*	
C5	0.1836 (4)	0.4445 (4)	0.2893 (2)	0.0670 (14)	
H5	0.1493	0.4444	0.3246	0.080*	
C3	0.3193 (4)	0.3866 (3)	0.2438 (3)	0.0720 (14)	
H3A	0.3773	0.3473	0.2489	0.086*	
C27	0.1302 (4)	1.0495 (3)	0.1514 (3)	0.0645 (14)	
H27	0.1558	1.1110	0.1623	0.077*	
C15	0.1682 (4)	0.7648 (3)	−0.1310 (2)	0.0622 (13)	
H15	0.2148	0.7895	−0.1540	0.075*	
C22	−0.2350 (4)	0.5316 (4)	0.1163 (3)	0.0779 (16)	
H22D	−0.2805	0.5035	0.0741	0.117*	
H22E	−0.2764	0.5598	0.1439	0.117*	
H22C	−0.1904	0.4833	0.1440	0.117*	
C4	0.2673 (5)	0.3872 (4)	0.2957 (3)	0.0826 (17)	
H4	0.2902	0.3478	0.3353	0.099*	
C26	0.0291 (4)	1.0369 (3)	0.1054 (2)	0.0570 (12)	
H26	−0.0113	1.0899	0.0864	0.068*	
C23	−0.2355 (4)	0.6871 (4)	0.0513 (3)	0.0736 (15)	
H23A	−0.2814	0.6600	0.0088	0.110*	
H23B	−0.1911	0.7339	0.0388	0.110*	
H23C	−0.2765	0.7169	0.0786	0.110*	
C18	0.4309 (4)	0.3987 (4)	0.1404 (3)	0.0895 (17)	
H18A	0.4171	0.3309	0.1336	0.107*	
H18B	0.4767	0.4085	0.1881	0.107*	
C33	−0.2833 (4)	0.9583 (4)	−0.0174 (3)	0.0921 (18)	
H33E	−0.3325	1.0074	−0.0388	0.138*	
H33D	−0.3091	0.9234	0.0163	0.138*	
H33C	−0.2747	0.9156	−0.0537	0.138*	
C19	0.4820 (5)	0.4348 (5)	0.0872 (4)	0.1229 (17)	
H19D	0.5439	0.3980	0.0899	0.184*	
H19E	0.5010	0.5006	0.0970	0.184*	
H19C	0.4342	0.4293	0.0404	0.184*	
C20	0.3736 (4)	0.8077 (4)	−0.0458 (3)	0.099 (2)	
H20C	0.3576	0.8665	−0.0730	0.119*	
H20B	0.3805	0.7568	−0.0777	0.119*	
C21	0.4742 (5)	0.8185 (5)	0.0139 (4)	0.1229 (17)	
H21D	0.5327	0.8221	−0.0056	0.184*	
H21B	0.4828	0.7644	0.0451	0.184*	
H21E	0.4710	0.8757	0.0401	0.184*	
O1W	0.3766 (3)	0.6483 (3)	0.1020 (2)	0.1109 (15)	
H1WA	0.3438	0.5975	0.1061	0.166*	
H1WB	0.3331	0.6857	0.0744	0.166*	
O7B	0.500 (3)	0.7112 (17)	0.2156 (14)	0.187 (8)	0.433 (10)
H7B	0.5110	0.7029	0.1769	0.281*	0.433 (10)
C34B	0.483 (2)	0.614 (2)	0.3126 (16)	0.226 (9)	0.433 (10)
H34H	0.5100	0.6487	0.3561	0.338*	0.433 (10)
H34G	0.4872	0.5466	0.3226	0.338*	0.433 (10)
H34C	0.4105	0.6310	0.2916	0.338*	0.433 (10)
C35B	0.5463 (16)	0.6375 (16)	0.2612 (12)	0.138 (5)	0.433 (10)
H35E	0.6168	0.6560	0.2880	0.166*	0.433 (10)
H35B	0.5515	0.5813	0.2335	0.166*	0.433 (10)
O7A	0.4612 (19)	0.7350 (11)	0.2408 (10)	0.187 (8)	0.567 (10)
H7A	0.4379	0.7212	0.1986	0.281*	0.567 (10)
C34A	0.5407 (18)	0.5896 (16)	0.2956 (15)	0.226 (9)	0.567 (10)
H34B	0.5435	0.5513	0.3367	0.338*	0.567 (10)
H34D	0.6049	0.6249	0.3035	0.338*	0.567 (10)
H34A	0.5317	0.5491	0.2549	0.338*	0.567 (10)
C35A	0.4496 (10)	0.6580 (10)	0.2828 (9)	0.138 (5)	0.567 (10)
H35A	0.3852	0.6245	0.2596	0.166*	0.567 (10)
H35C	0.4440	0.6815	0.3282	0.166*	0.567 (10)

### Atomic displacement parameters (Å^2^)

**Table:**

	*U*^11^	*U*^22^	*U*^33^	*U*^12^	*U*^13^	*U*^23^
Co1	0.0447 (3)	0.0301 (3)	0.0365 (3)	−0.0004 (3)	0.0113 (2)	−0.0034 (2)
O1	0.0476 (17)	0.0347 (14)	0.0375 (15)	0.0035 (12)	0.0118 (13)	−0.0009 (12)
O2	0.0439 (17)	0.0405 (15)	0.0408 (16)	−0.0030 (13)	0.0109 (13)	0.0034 (12)
O3	0.0494 (18)	0.0260 (14)	0.0538 (17)	−0.0013 (12)	0.0101 (14)	−0.0069 (12)
O4	0.056 (2)	0.066 (2)	0.071 (2)	0.0214 (16)	0.0230 (18)	0.0092 (17)
O5	0.054 (2)	0.067 (2)	0.077 (2)	−0.0061 (17)	0.0308 (19)	0.0121 (17)
O6	0.059 (2)	0.0381 (16)	0.070 (2)	0.0076 (15)	0.0171 (17)	0.0010 (14)
N1	0.046 (2)	0.0384 (18)	0.0389 (19)	0.0002 (16)	0.0124 (16)	−0.0070 (16)
N2	0.044 (2)	0.0275 (16)	0.0386 (19)	0.0012 (15)	0.0144 (16)	−0.0034 (14)
O7	0.0524 (18)	0.0415 (17)	0.0427 (16)	−0.0020 (14)	0.0058 (14)	−0.0032 (13)
C1	0.045 (3)	0.035 (2)	0.037 (2)	−0.0011 (19)	0.006 (2)	0.0004 (18)
C17	0.055 (3)	0.025 (2)	0.040 (2)	0.0093 (18)	0.017 (2)	0.0008 (17)
C30	0.051 (3)	0.034 (2)	0.044 (2)	−0.0012 (19)	0.024 (2)	−0.0052 (18)
C10	0.049 (3)	0.037 (2)	0.045 (2)	−0.0064 (19)	0.011 (2)	−0.0042 (19)
C12	0.058 (3)	0.024 (2)	0.038 (2)	0.0021 (18)	0.014 (2)	0.0007 (16)
C6	0.054 (3)	0.044 (2)	0.041 (2)	0.005 (2)	0.015 (2)	0.005 (2)
C11	0.047 (3)	0.028 (2)	0.039 (2)	0.0034 (18)	0.003 (2)	−0.0072 (17)
C32	0.077 (4)	0.047 (3)	0.074 (3)	0.021 (3)	0.034 (3)	0.015 (2)
C16	0.054 (3)	0.036 (2)	0.057 (3)	0.005 (2)	0.022 (2)	0.004 (2)
C25	0.056 (3)	0.035 (2)	0.051 (3)	0.002 (2)	0.027 (2)	−0.0046 (19)
C8	0.048 (3)	0.052 (3)	0.051 (2)	0.001 (2)	0.022 (2)	−0.006 (2)
C7	0.061 (3)	0.053 (3)	0.038 (2)	−0.008 (2)	0.021 (2)	−0.005 (2)
C29	0.061 (3)	0.042 (2)	0.041 (2)	−0.005 (2)	0.019 (2)	−0.007 (2)
C2	0.053 (3)	0.047 (3)	0.058 (3)	0.002 (2)	0.013 (2)	0.006 (2)
C13	0.080 (3)	0.038 (2)	0.044 (3)	0.005 (2)	0.014 (2)	0.002 (2)
C28	0.065 (3)	0.043 (3)	0.068 (3)	−0.018 (2)	0.026 (3)	−0.014 (2)
C14	0.090 (4)	0.048 (3)	0.044 (3)	0.006 (3)	0.020 (3)	0.005 (2)
C9	0.040 (3)	0.048 (3)	0.059 (3)	−0.002 (2)	0.017 (2)	−0.007 (2)
C31	0.055 (3)	0.055 (3)	0.042 (2)	−0.012 (2)	0.007 (2)	−0.010 (2)
C5	0.076 (4)	0.076 (3)	0.050 (3)	0.006 (3)	0.019 (3)	0.020 (3)
C3	0.072 (4)	0.066 (3)	0.074 (3)	0.022 (3)	0.013 (3)	0.024 (3)
C27	0.087 (4)	0.037 (3)	0.075 (3)	−0.018 (3)	0.033 (3)	−0.020 (2)
C15	0.089 (4)	0.052 (3)	0.057 (3)	0.008 (3)	0.039 (3)	0.013 (2)
C22	0.067 (4)	0.089 (4)	0.089 (4)	−0.028 (3)	0.042 (3)	−0.018 (3)
C4	0.089 (4)	0.086 (4)	0.067 (4)	0.020 (3)	0.013 (3)	0.044 (3)
C26	0.075 (4)	0.031 (2)	0.075 (3)	−0.002 (2)	0.037 (3)	−0.003 (2)
C23	0.064 (3)	0.078 (4)	0.071 (3)	0.014 (3)	0.007 (3)	−0.014 (3)
C18	0.073 (4)	0.087 (4)	0.111 (5)	0.029 (3)	0.030 (4)	0.005 (4)
C33	0.064 (4)	0.086 (4)	0.112 (5)	0.017 (3)	0.001 (3)	0.011 (4)
C19	0.069 (3)	0.137 (4)	0.172 (5)	−0.002 (3)	0.050 (3)	−0.001 (4)
C20	0.079 (4)	0.098 (5)	0.134 (5)	0.000 (4)	0.055 (4)	0.016 (4)
C21	0.069 (3)	0.137 (4)	0.172 (5)	−0.002 (3)	0.050 (3)	−0.001 (4)
O1W	0.074 (3)	0.105 (3)	0.148 (4)	0.000 (2)	0.022 (3)	0.045 (3)
O7B	0.201 (19)	0.138 (10)	0.179 (14)	−0.046 (8)	−0.017 (10)	−0.026 (10)
C34B	0.20 (3)	0.23 (2)	0.21 (2)	0.105 (17)	−0.010 (18)	−0.023 (16)
C35B	0.119 (10)	0.118 (11)	0.142 (12)	0.006 (9)	−0.021 (9)	−0.004 (8)
O7A	0.201 (19)	0.138 (10)	0.179 (14)	−0.046 (8)	−0.017 (10)	−0.026 (10)
C34A	0.20 (3)	0.23 (2)	0.21 (2)	0.105 (17)	−0.010 (18)	−0.023 (16)
C35A	0.119 (10)	0.118 (11)	0.142 (12)	0.006 (9)	−0.021 (9)	−0.004 (8)

### Geometric parameters (Å, °)

**Table:**

Co1—O3	1.882 (2)	C9—C22	1.525 (6)
Co1—O2	1.885 (3)	C9—C23	1.531 (6)
Co1—O1	1.891 (2)	C31—H31	0.9300
Co1—N2	1.891 (3)	C5—C4	1.348 (6)
Co1—N1	1.910 (3)	C5—H5	0.9300
Co1—O7	1.923 (3)	C3—C4	1.382 (7)
O1—C1	1.313 (4)	C3—H3A	0.9300
O2—C17	1.311 (4)	C27—C26	1.400 (6)
O3—C30	1.294 (4)	C27—H27	0.9300
O4—C2	1.378 (5)	C15—H15	0.9300
O4—C18	1.436 (5)	C22—H22D	0.9600
O5—C16	1.369 (5)	C22—H22E	0.9600
O5—C20	1.414 (6)	C22—H22C	0.9600
O6—C25	1.358 (5)	C4—H4	0.9300
O6—C32	1.437 (5)	C26—H26	0.9300
N1—C7	1.295 (5)	C23—H23A	0.9600
N1—C8	1.456 (5)	C23—H23B	0.9600
N2—C11	1.288 (4)	C23—H23C	0.9600
N2—C10	1.468 (5)	C18—C19	1.486 (8)
O7—C31	1.247 (5)	C18—H18A	0.9700
C1—C6	1.405 (5)	C18—H18B	0.9700
C1—C2	1.421 (5)	C33—H33E	0.9600
C17—C12	1.404 (5)	C33—H33D	0.9600
C17—C16	1.423 (5)	C33—H33C	0.9600
C30—C29	1.410 (6)	C19—H19D	0.9600
C30—C25	1.440 (5)	C19—H19E	0.9600
C10—C9	1.538 (5)	C19—H19C	0.9600
C10—H10A	0.9700	C20—C21	1.514 (8)
C10—H10B	0.9700	C20—H20C	0.9700
C12—C13	1.418 (5)	C20—H20B	0.9700
C12—C11	1.427 (5)	C21—H21D	0.9600
C6—C5	1.397 (6)	C21—H21B	0.9600
C6—C7	1.429 (6)	C21—H21E	0.9600
C11—H11	0.9300	O1W—H1WA	0.8508
C32—C33	1.487 (6)	O1W—H1WB	0.8508
C32—H32A	0.9700	O7B—C35B	1.390 (10)
C32—H32B	0.9700	O7B—H7B	0.8202
C16—C15	1.379 (6)	O7B—H7A	0.8084
C25—C26	1.361 (5)	C34B—C35B	1.526 (10)
C8—C9	1.561 (5)	C34B—H34H	0.9600
C8—H8C	0.9700	C34B—H34G	0.9600
C8—H8B	0.9700	C34B—H34C	0.9600
C7—H7	0.9300	C35B—H35E	0.9700
C29—C31	1.407 (6)	C35B—H35B	0.9700
C29—C28	1.434 (5)	O7A—C35A	1.392 (9)
C2—C3	1.360 (6)	O7A—H7A	0.8200
C13—C14	1.353 (6)	C34A—C35A	1.508 (9)
C13—H13	0.9300	C34A—H34B	0.9600
C28—C27	1.338 (6)	C34A—H34D	0.9600
C28—H28	0.9300	C34A—H34A	0.9600
C14—C15	1.392 (6)	C35A—H35A	0.9700
C14—H14	0.9300	C35A—H35C	0.9700
			
O3—Co1—O2	88.56 (11)	C23—C9—C10	110.6 (3)
O3—Co1—O1	175.99 (11)	C22—C9—C8	109.4 (4)
O2—Co1—O1	87.52 (11)	C23—C9—C8	109.1 (3)
O3—Co1—N2	86.00 (12)	C10—C9—C8	110.5 (3)
O2—Co1—N2	95.04 (12)	O7—C31—C29	128.4 (4)
O1—Co1—N2	93.50 (11)	O7—C31—H31	115.8
O3—Co1—N1	91.77 (13)	C29—C31—H31	115.8
O2—Co1—N1	176.57 (12)	C4—C5—C6	120.8 (5)
O1—Co1—N1	92.20 (12)	C4—C5—H5	119.6
N2—Co1—N1	88.39 (13)	C6—C5—H5	119.6
O3—Co1—O7	93.96 (11)	C2—C3—C4	120.5 (5)
O2—Co1—O7	85.60 (11)	C2—C3—H3A	119.8
O1—Co1—O7	86.58 (11)	C4—C3—H3A	119.8
N2—Co1—O7	179.36 (13)	C28—C27—C26	121.7 (4)
N1—Co1—O7	90.97 (12)	C28—C27—H27	119.2
C1—O1—Co1	123.2 (2)	C26—C27—H27	119.2
C17—O2—Co1	125.7 (2)	C16—C15—C14	120.4 (4)
C30—O3—Co1	123.8 (2)	C16—C15—H15	119.8
C2—O4—C18	118.0 (4)	C14—C15—H15	119.8
C16—O5—C20	117.8 (4)	C9—C22—H22D	109.5
C25—O6—C32	118.3 (3)	C9—C22—H22E	109.5
C7—N1—C8	118.8 (4)	H22D—C22—H22E	109.5
C7—N1—Co1	123.3 (3)	C9—C22—H22C	109.5
C8—N1—Co1	117.6 (3)	H22D—C22—H22C	109.5
C11—N2—C10	117.6 (3)	H22E—C22—H22C	109.5
C11—N2—Co1	123.9 (3)	C5—C4—C3	120.4 (4)
C10—N2—Co1	118.4 (2)	C5—C4—H4	119.8
C31—O7—Co1	122.5 (3)	C3—C4—H4	119.8
O1—C1—C6	124.4 (4)	C25—C26—C27	120.5 (4)
O1—C1—C2	118.5 (4)	C25—C26—H26	119.8
C6—C1—C2	116.9 (4)	C27—C26—H26	119.8
O2—C17—C12	125.1 (4)	C9—C23—H23A	109.5
O2—C17—C16	118.3 (4)	C9—C23—H23B	109.5
C12—C17—C16	116.6 (4)	H23A—C23—H23B	109.5
O3—C30—C29	125.5 (4)	C9—C23—H23C	109.5
O3—C30—C25	117.4 (4)	H23A—C23—H23C	109.5
C29—C30—C25	117.1 (4)	H23B—C23—H23C	109.5
N2—C10—C9	113.7 (3)	O4—C18—C19	108.7 (5)
N2—C10—H10A	108.8	O4—C18—H18A	110.0
C9—C10—H10A	108.8	C19—C18—H18A	110.0
N2—C10—H10B	108.8	O4—C18—H18B	110.0
C9—C10—H10B	108.8	C19—C18—H18B	110.0
H10A—C10—H10B	107.7	H18A—C18—H18B	108.3
C17—C12—C13	120.6 (4)	C32—C33—H33E	109.5
C17—C12—C11	121.6 (3)	C32—C33—H33D	109.5
C13—C12—C11	117.3 (4)	H33E—C33—H33D	109.5
C5—C6—C1	120.3 (4)	C32—C33—H33C	109.5
C5—C6—C7	119.0 (4)	H33E—C33—H33C	109.5
C1—C6—C7	120.4 (4)	H33D—C33—H33C	109.5
N2—C11—C12	127.0 (4)	C18—C19—H19D	109.5
N2—C11—H11	116.5	C18—C19—H19E	109.5
C12—C11—H11	116.5	H19D—C19—H19E	109.5
O6—C32—C33	107.2 (4)	C18—C19—H19C	109.5
O6—C32—H32A	110.3	H19D—C19—H19C	109.5
C33—C32—H32A	110.3	H19E—C19—H19C	109.5
O6—C32—H32B	110.3	O5—C20—C21	107.0 (5)
C33—C32—H32B	110.3	O5—C20—H20C	110.3
H32A—C32—H32B	108.5	C21—C20—H20C	110.3
O5—C16—C15	124.9 (4)	O5—C20—H20B	110.3
O5—C16—C17	113.6 (4)	C21—C20—H20B	110.3
C15—C16—C17	121.5 (4)	H20C—C20—H20B	108.6
O6—C25—C26	126.4 (4)	C20—C21—H21D	109.5
O6—C25—C30	112.8 (3)	C20—C21—H21B	109.5
C26—C25—C30	120.7 (4)	H21D—C21—H21B	109.5
N1—C8—C9	110.7 (3)	C20—C21—H21E	109.5
N1—C8—H8C	109.5	H21D—C21—H21E	109.5
C9—C8—H8C	109.5	H21B—C21—H21E	109.5
N1—C8—H8B	109.5	H1WA—O1W—H1WB	107.5
C9—C8—H8B	109.5	C35B—O7B—H7B	109.1
H8C—C8—H8B	108.1	C35B—O7B—H7A	127.3
N1—C7—C6	126.6 (4)	H7B—O7B—H7A	94.1
N1—C7—H7	116.7	C35B—C34B—H34H	109.5
C6—C7—H7	116.7	C35B—C34B—H34G	109.5
C31—C29—C30	120.9 (4)	H34H—C34B—H34G	109.5
C31—C29—C28	118.5 (4)	C35B—C34B—H34C	109.5
C30—C29—C28	120.5 (4)	H34H—C34B—H34C	109.5
C3—C2—O4	124.8 (4)	H34G—C34B—H34C	109.5
C3—C2—C1	121.1 (4)	O7B—C35B—C34B	111.1 (10)
O4—C2—C1	114.0 (4)	O7B—C35B—H35E	109.4
C14—C13—C12	120.8 (4)	C34B—C35B—H35E	109.4
C14—C13—H13	119.6	O7B—C35B—H35B	109.4
C12—C13—H13	119.6	C34B—C35B—H35B	109.4
C27—C28—C29	119.5 (4)	H35E—C35B—H35B	108.0
C27—C28—H28	120.3	C35A—O7A—H7A	109.9
C29—C28—H28	120.3	O7A—C35A—C34A	111.9 (10)
C13—C14—C15	119.9 (4)	O7A—C35A—H35A	109.2
C13—C14—H14	120.0	C34A—C35A—H35A	109.2
C15—C14—H14	120.0	O7A—C35A—H35C	109.2
C22—C9—C23	111.4 (4)	C34A—C35A—H35C	109.2
C22—C9—C10	105.9 (3)	H35A—C35A—H35C	107.9
			
O2—Co1—O1—C1	142.6 (3)	O2—C17—C16—O5	−1.2 (5)
N2—Co1—O1—C1	−122.5 (3)	C12—C17—C16—O5	179.9 (3)
N1—Co1—O1—C1	−34.0 (3)	O2—C17—C16—C15	178.8 (4)
O7—Co1—O1—C1	56.8 (3)	C12—C17—C16—C15	−0.1 (5)
O3—Co1—O2—C17	−76.0 (3)	C32—O6—C25—C26	−3.4 (6)
O1—Co1—O2—C17	103.2 (3)	C32—O6—C25—C30	176.5 (3)
N2—Co1—O2—C17	9.9 (3)	O3—C30—C25—O6	1.2 (5)
O7—Co1—O2—C17	−170.1 (3)	C29—C30—C25—O6	−176.7 (3)
O2—Co1—O3—C30	−62.5 (3)	O3—C30—C25—C26	−178.9 (4)
N2—Co1—O3—C30	−157.7 (3)	C29—C30—C25—C26	3.2 (6)
N1—Co1—O3—C30	114.0 (3)	C7—N1—C8—C9	−103.9 (4)
O7—Co1—O3—C30	22.9 (3)	Co1—N1—C8—C9	69.2 (4)
O3—Co1—N1—C7	−156.5 (3)	C8—N1—C7—C6	166.9 (4)
O1—Co1—N1—C7	24.1 (3)	Co1—N1—C7—C6	−5.8 (6)
N2—Co1—N1—C7	117.5 (3)	C5—C6—C7—N1	173.3 (4)
O7—Co1—N1—C7	−62.6 (3)	C1—C6—C7—N1	−12.9 (6)
O3—Co1—N1—C8	30.7 (3)	O3—C30—C29—C31	−3.1 (6)
O1—Co1—N1—C8	−148.7 (3)	C25—C30—C29—C31	174.6 (4)
N2—Co1—N1—C8	−55.3 (3)	O3—C30—C29—C28	−179.5 (4)
O7—Co1—N1—C8	124.7 (3)	C25—C30—C29—C28	−1.8 (6)
O3—Co1—N2—C11	74.6 (3)	C18—O4—C2—C3	−5.3 (7)
O2—Co1—N2—C11	−13.6 (3)	C18—O4—C2—C1	172.6 (4)
O1—Co1—N2—C11	−101.4 (3)	O1—C1—C2—C3	−176.3 (4)
N1—Co1—N2—C11	166.5 (3)	C6—C1—C2—C3	0.1 (6)
O3—Co1—N2—C10	−101.8 (3)	O1—C1—C2—O4	5.6 (5)
O2—Co1—N2—C10	170.0 (2)	C6—C1—C2—O4	−178.0 (4)
O1—Co1—N2—C10	82.2 (3)	C17—C12—C13—C14	0.8 (6)
N1—Co1—N2—C10	−9.9 (3)	C11—C12—C13—C14	−171.5 (4)
O3—Co1—O7—C31	−20.5 (3)	C31—C29—C28—C27	−177.0 (4)
O2—Co1—O7—C31	67.8 (3)	C30—C29—C28—C27	−0.6 (6)
O1—Co1—O7—C31	155.5 (3)	C12—C13—C14—C15	−0.4 (6)
N1—Co1—O7—C31	−112.3 (3)	N2—C10—C9—C22	−173.8 (3)
Co1—O1—C1—C6	26.2 (5)	N2—C10—C9—C23	65.4 (4)
Co1—O1—C1—C2	−157.7 (3)	N2—C10—C9—C8	−55.5 (4)
Co1—O2—C17—C12	−1.3 (5)	N1—C8—C9—C22	107.1 (4)
Co1—O2—C17—C16	179.9 (2)	N1—C8—C9—C23	−130.9 (4)
Co1—O3—C30—C29	−15.0 (5)	N1—C8—C9—C10	−9.2 (5)
Co1—O3—C30—C25	167.2 (2)	Co1—O7—C31—C29	10.0 (6)
C11—N2—C10—C9	−112.7 (4)	C30—C29—C31—O7	5.7 (7)
Co1—N2—C10—C9	64.0 (4)	C28—C29—C31—O7	−177.9 (4)
O2—C17—C12—C13	−179.3 (3)	C1—C6—C5—C4	0.5 (7)
C16—C17—C12—C13	−0.5 (5)	C7—C6—C5—C4	174.2 (5)
O2—C17—C12—C11	−7.4 (6)	O4—C2—C3—C4	178.3 (5)
C16—C17—C12—C11	171.4 (3)	C1—C2—C3—C4	0.5 (7)
O1—C1—C6—C5	175.6 (4)	C29—C28—C27—C26	1.6 (7)
C2—C1—C6—C5	−0.5 (6)	O5—C16—C15—C14	−179.6 (4)
O1—C1—C6—C7	2.0 (6)	C17—C16—C15—C14	0.5 (6)
C2—C1—C6—C7	−174.2 (4)	C13—C14—C15—C16	−0.2 (7)
C10—N2—C11—C12	−174.1 (3)	C6—C5—C4—C3	0.1 (8)
Co1—N2—C11—C12	9.5 (5)	C2—C3—C4—C5	−0.6 (8)
C17—C12—C11—N2	2.9 (6)	O6—C25—C26—C27	177.6 (4)
C13—C12—C11—N2	175.1 (4)	C30—C25—C26—C27	−2.3 (6)
C25—O6—C32—C33	−174.0 (4)	C28—C27—C26—C25	−0.2 (7)
C20—O5—C16—C15	−11.7 (6)	C2—O4—C18—C19	−165.4 (4)
C20—O5—C16—C17	168.2 (4)	C16—O5—C20—C21	−169.8 (4)

### Hydrogen-bond geometry (Å, °)

**Table:**

*D*—H···*A*	*D*—H	H···*A*	*D*···*A*	*D*—H···*A*
O1W—H1WA···O1	0.85	2.51	3.182 (5)	137
O1W—H1WA···O4	0.85	2.15	2.936 (5)	154
O1W—H1WB···O2	0.85	2.21	2.883 (5)	136
O1W—H1WB···O5	0.85	2.18	2.952 (5)	151
O7A—H7A···O1W	0.82	2.10	2.899 (19)	164
C8—H8C···O3	0.97	2.31	2.829 (5)	113
